# Special Issue “Laser Technologies in Metal-Based Materials”

**DOI:** 10.3390/ma16134511

**Published:** 2023-06-21

**Authors:** Alina Manshina

**Affiliations:** Institute of Chemistry, Saint-Petersburg State University, 26 Universitetskii Prospect, Saint-Petersburg 198504, Russia; a.manshina@spbu.ru

The first publication, analyzing the prospects for the use of laser radiation, was published under the authorship of the American physicist Arthur Shawlow in November 1960 (Schawlow, A. L. Bell Lab. Rec., November, 403 (1960)) immediately after the creation of the first laser by Theodor Meiman on 16 May 1960. Later, Arthur Shawlow received the Nobel Prize. Subsequently, many brilliant scientists (A. Zeveil, V.S. Letokhov, N.V. Karlov, and many others) joined the topic of laser-induced processes, which ensured rapid progress in this area [1,2,3,4,5,6,7]. As a result, new directions in chemistry and physics have been formed—laser chemistry and laser physics, which continue to be a dynamically developing science. These laser-related directions consider the fundamental issues of the synthesis/transformation of substances and the problems of high precision and highly controlled laser technologies. Insightful publications of the late 20th century reporting on original ideas of laser irradiation use for various processes of materials transformation and fabrication [8,9,10] turned into extensive areas related to laser technologies since the beginning of the 21st century.

This Special Issue aims to bring the fields of laser technologies and metal nanostructures together for both benefits. We consider different aspects of laser technologies for fabrication of metal-based functional nanomaterials here, as numerous modern instruments and devices are based on processes related to metal nanostructures. It should be noted that the laser effect on a material can initiate physical phenomena (heating, phase transitions, etc.) and/or chemical phenomena (oxidation, reduction, chemical transformations). Thus, the articles of the current Special issue harmoniously combine physical and chemical phenomena and offer advanced laser technologies to modern society.

Regarding publications in laser-induced physical processes, one can find the article by A. V. Agapovichev et al. on selective laser melting to produce Ni-Cr-Al-Ti-Based Superalloy [11]. The authors of the article present sintering processes by pulsed nanosecond laser for obtaining aerosol agglomerates of Pt, Au, and Ag NPs [12]. The interesting combination of processes of laser-induced surface texturing simultaneously with laser-induced anchoring of silver NPs from colloidal solution is discussed by Jakub Siegel et al. in [13]. Such textured polymer surfaces decorated with Ag NPs can be prospective antimicrobial coatings. Another example of laser-induced physical phenomena is laser shock peening, demonstrating significantly improving the fretting fatigue life of TC11 titanium alloy [14]. In the article by Piotr Kupracz et al. [15] laser re-solidification was demonstrated as an approach for the modulation of morphology and structure of metal-decorated TiO_2_ nanotubes to obtain visible light harvesting.

Interesting advanced approaches for creating nanostructured metal materials with various functionality were presented in laser-induced chemical processes. Thus, laser ablation of monocrystalline silicon in isopropanol containing AgNO_3_ allowed the single-step formation of Ag-decorated Si microspheres with SERS performance [16]. Here, the physical process of laser ablation is accompanied by the chemical process of Ag NPs formation onto ablated Si species. Femtosecond laser reductive sintering allowed for obtaining high-purity Cu patterns from CuO NPs inks [17]. At the same time, a variant of selective laser reductive sintering created copper and nickel microsensors for non-enzymatic glucose detection [18]. Highly controllable decoration of substrates by plasmonic Ag, Pt NPs with uniform or periodic NPs distribution was demonstrated due to laser-induced deposition [19]. This laser-induced process is based on the photodecomposition of metal-containing precursors and following redox processes onto the substrate surface. Interestingly, a similar process can be realized as a laser-induced thermal process resulting in composite materials based on iridium, gold, and platinum [20].
